# An Age and Activity Algorithm for Treatment of Type II SLAP Tears

**DOI:** 10.2174/1874325001812010271

**Published:** 2018-07-31

**Authors:** Michael D. Charles, David R. Christian, Brian J. Cole

**Affiliations:** Department of Orthopaedic Surgery, Rush University Medical Center, Chicago, IL 60612, USA

**Keywords:** Type II SLAP tear, Superior labrum-biceps complex, SLAP repair, Biceps tenodesis, Non-operative treatment, Overhead athlete

## Abstract

**Background:**

Type II SLAP tears predominantly occur in males between their third and fifth decades of life. The mechanism of injury is often repeated overheard activity but can also occur due to direct compression loads and traction injuries. The treatment options have changed over the years and include non-operative therapy, direct labral-biceps complex repair, and labral debridement with biceps tenodesis or tenotomy.

**Objective:**

To review the existing literature on the management of Type II SLAP tears and provide clinical recommendations based on patient age and activity level.

**Methods:**

A review of the existing literature through October 2017 investigating the management of Type II SLAP tears was performed. Emphasis was placed on distinguishing the outcomes based on age and activity level to provide an appropriate treatment algorithm.

**Results:**

Patients with Type II SLAP tears should first be trialed with non-operative management and many patients will have a successful result with ability to return to their respective sports or activities. Surgical management should be considered if non-operative management does not provide symptomatic relief. Young, athletic, or high-demand patients should be treated with a SLAP repair while biceps tenodesis should be considered for older or worker’s compensation patients. Patients undergoing revision surgery for a failed SLAP repair should be managed with biceps tenodesis.

**Conclusion:**

Type II SLAP tears remain a difficult pathology to manage clinically, but the treatment indications are narrowing. The age and activity algorithm described in this review provides an effective method of managing this complex clinical condition.

## 1 INTRODUCTION

Tears of the Superior Labrum-Biceps Complex (SLBC) were first described by Andrews *et al* [1] as Superior Labrum Anterior to Posterior (SLAP) tears. Snyder *et al* [2] organized these SLAP tears into four major types: 1) Labral fraying with intact bicep anchor; 2) Labral fraying with detachment of bicep anchor; 3) Bucket handle tear of labrum with intact bicep anchor; and 4) Bucket handle tear of the labrum that extends into the bicep anchor. These four types of SLAP tears were later expanded by Maffet *et al* [3]. SLAP tears are not common injuries to the shoulder, but often result from repetitive overhead activity due to a “Peel-back Mechanism,” [4] direct compression loads, or occasionally traction injuries [5]. These lesions predominantly occur in males and are often treated in the third and fifth decades of life [6]. SLAP tears can be difficult to diagnosis as a patient’s history can be mixed, with the most common complaint of pain [5]. Overhead throwers may mention a typical history of loss of velocity or control [7]. Physical examination is also unable to diagnose SLAP tears with consistency [8-11] as most exam maneuvers may suggest but not confirm a SLAP tear. Part of the difficulty is that while SLAP tears do occur in isolation, they can also be found with concomitant pathology including rotator cuff tears, labral tears, Acromio-clavicular joint pathology, and impingement in up to 88% of cases [12, 13]. Magnetic Resonance Imaging (MRI) has been useful in identifying SLAP lesion [14] despite multiple anatomical variants [15], but MRI arthrogram remains the gold standard for imaging.

**Fig. (1):**
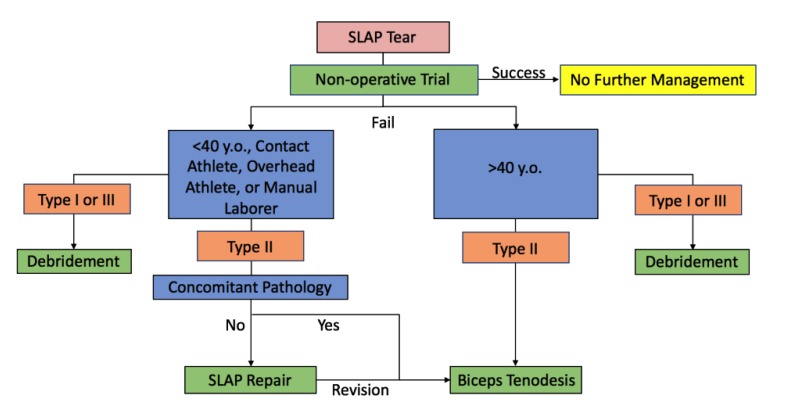
**Algorithm for Management of SLAP Tears.** Red represents overarching observed pathology. Green represents treatment options. Blue represents patient factors involved in decision making. Orange represents types of SLAP tears. SLAP tears = superior labrum anterior to posterior tears.

As these lesions became better defined and imaging quality improved there was an increase in diagnosis [16] and surgical treatment of slap lesions [6, 17] until the late 2000’s. However, as our understanding of the pathology, outcomes, and complications improved there has been a decreased frequency and change in approach to the surgical treatment of SLAP tears [18, 19]. Much of this change is centered upon the treatment of Type II SLAP tears. While the general consensus on the treatment of Type I and III tears centers on debridement [5, 20, 21], treatment of type II tears continues to be studied and modified. To date, numerous studies have examined treatment of type II SLAP tears [20, 22-37] including multiple systematic and database reviews [6, 16, 18, 19, 38-41]. Current treatment options for Type II SLAP tears include non-operative, direct labral-bicep complex repair, and debridement with tenodesis or tenotomy. The primary treatment in type II SLAP tears was originally direct repair, but has undergone a slow development of a treatment algorithm that is based on the patient’s age and activity level for most surgeons as reflected in the most recent ABOS board review data [19]. A database of our senior author (BJC) and his colleagues have reflected a growing use of bicep tenodesis for SLAP tears and other shoulder pathologies [42]. A recent randomized control trial out of Norway looked at outcomes between sham surgery, labral repair, and bicep tenodesis for Type II SLAP tears [36]. The authors report no significant difference between the three groups in terms of outcomes. These results need to be tempered as patients were organized as intent to treat and 14 of 39 (35.9%) of sham patients required repeat surgeries (either for repair or tenodesis), while the tenodesis group had 6 repeat surgeries (2 capsular releases, 3 labral repair, and 1 AC joint resection) and the repair group had only 4 repeat surgeries (3 bicep tenodesis and 1 AC joint resection) [36]. Schrøeder *et al* recognizes the influence of these cross over patients and concludes that SLAP tears may be overtreated and requires more narrowed indications in the case of repair. In that light, our paper explains the senior author’s indications and reasoning behind the treatment for each treatment group (Fig. **1**).

### 2 NON-OPERATIVE MANAGEMENT

In most cases, all patients should first be trialed with non-operative treatment. This decision-making is in the context that incidental labral abnormalities are common by MRI, and the natural history of the neglected-labral tear is not associated with the development of other relevant pathology that can otherwise be prevented with early treatment. In other words, benign neglect is acceptable if the patient can be made to tolerate their symptoms or to become asymptomatic with normal function. Non-operative treatment has shown success in groups including high-level athletes [43-45]. Edwards *et al* [43] performed a retrospective review of people treated non-operatively including athletes. While the paper is limited by poor response (39 out of 371 eligible patients) their findings demonstrated 49% of patients were deemed successfully treated with posterior capsular stretching and scapular stabilization programs. Those that were successful saw significant improvements in ASES (58.5 to 84.7) and VAS (4.5 to 2.1) and all athletes were able to return to their sport. Including the failure 71% of athletes returned to their sport with non-operative treatment and 67% for overhead athletes [43]. Jang *et al* [45] investigated possible risk factors for people who would fail non-operative treatment. In their retrospective study of 63 patients, 71% of patients had successful non-operative treatment at 21 months. Multivariate analysis demonstrated that patients with a history of trauma (OR 9.8), positive compression test (OR 8.8), and participation in overhead activities (OR 19.1) were more likely to fail non-operative treatment [45]. Non-operative management may have even a small but less successful role in elite overhead athletes. A retrospective review of a single major league baseball organization’s players demonstrated a return to play rate of 40% for pitchers and a return to previous level of play of only 22% with non-operative treatment. Despite a high failure rate, non-operative treatment did not differ much from surgical interventions, which saw a return to play rate of 48% and a return to previous level of play of 7% [44]. Of note, positional players fared much better with operative treatment, returning to play in 85% of cases versus 39% of those treated non-operatively, and those with concomitant partial cuff tears fared worse [44]. While successful non-operative treatment ranges from 40% to 70%, it should be at least trialed in most patient populations including elite throwers especially given the heterogeneous outcome in this population following surgical intervention.

### 3 YOUNG, ATHLETIC PATIENTS AND OVERHEAD THROWERS

Repetitive overhead motion, especially amongst throwers is believed to be one of the primary causes of SLAP tears. The biological adaptations in muscular and ligamentous anatomy in an overhead thrower was outlined by Burkhart *et al* [7] including the series of events that lead to the “peel back” mechanism that predisposes throwers to SLAP tears [4]. While not clearly defined, the role of the bicep tendon has been considered a vestigial organ [46] and primarily a pain generator for a dysfunctional shoulder [47]. Cadaveric studies, however, have pointed to the superior labrum and the long head of the bicep as an important glenohumeral stabilizer [48-52], especially in overhead throwers [53]. These studies however, had varying lesion size with significant but small changes in glenohumeral motion that may not be clinically significant and do not account for additional factors for glenohumeral motion and stability [46, 54].

Despite conflicting cadaveric studies, young active patients, particularly overhead throwers, have been deemed the best candidates for repair of type II SLAP tears. Systematic reviews have demonstrated that patients who are younger athletes have better outcome scores and more reliable return to sport following SLAP repair [26, 55]. Sayde *et al* [55] demonstrated 83% of patients reporting good to excellent results and 73% of athletes (63% overhead athletes) returned to their previous level of play. Kim *et al* [32] and Ide *et al* [56] reported excellent outcomes in 79% and 75% of their young (<40) athletic cohorts, respectively. Studies of athletic populations have consistently shown that overhead athletes do not have the same return to sport rates as non-overhead athletes despite both groups having excellent outcomes [26, 28, 32, 55-58]. Friel *et al* with the senior author (BJC) [28] exemplified this with excellent functional outcome scores, but five of the thirteen overhead athletes failed to return to previous level. Similar disparities in return to sport rates were found in larger studies by Kim *et al* [32] and Ide *et al* [56]. An exception to this trend was Morgan *et al* [57] who reviewed 102 patients (53 overhead athletes) and found that 87% had excellent one-year post-operative outcomes based on their UCLA shoulder scores. More specifically, of the 44 pitchers, 37 (84%) of them reported excellent outcomes at one year and only seven (all had partial cuff tears) subjectively did not return to their previous level of performance [57].

**Fig. (2):**
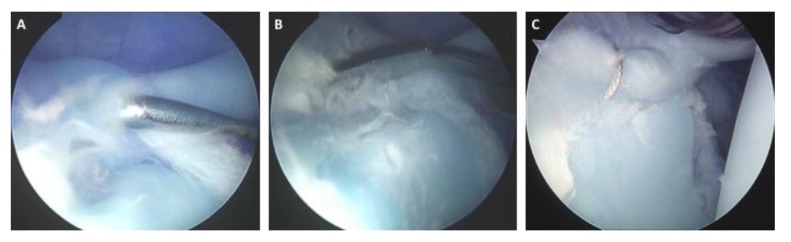
**Repair of a Type II SLAP Tear.** Intraoperative arthroscopic images of a right shoulder depicting A) a Type II SLAP tear with an intact long head of the biceps, B) elevation of the labrum from the superior glenoid using a Bankart elevator, and C) SLAP repair.

Elite level throwers are a population that has been closely studied. Baseball players, specifically pitchers, are susceptible to SLAP tears and have the lowest return to sport outcomes [58]. While overall shoulder scores improve with surgical treatment of Type II SLAP tears, the return to play outcome is significantly lower then other athletic populations [44, 59]. Smith *et al* [59] found that only 62.5% of Major League Baseball pitchers were able to return to the major league level, and only 54% returned to their previous level of performance. This is still an improvement compared to non-operative outcomes report by Fedoriw *et al* [44]. Military populations also tend to have worse outcomes then the general population. A study of 179 military patients with a mean follow up of 40 months saw significant improvement in their outcome scores, but 66 (36.8%) patients had failed SLAP repair requiring either medical discharge or revision surgery [34]. Age greater then thirty-six was the only risk factor for failure on sub group analysis. Due to the excellent outcomes, we recommend SLAP repair for most young and athletic patients who have no overlapping biceps pain and who have failed non-surgical intervention (Fig. **2**). At some point, we will often utilize selective injections as a pre-operative diagnostic tool. Immediate but temporary relief with a local anesthetic injection into the glenhohumeral joint in the absence of biceps pain or provocative symptoms in the biceps with physical examination is relatively specific to labral pathology in the absence of any other identifiable pain generator.

Tenodesis in a younger athletic patient may have a limited role in treating this patient group. Bicep tenodesis has proven effective in revision SLAP tears [23, 60-62]. McCormick *et al* [61] reviewed 42 patients with failed SLAP repairs, following tenodesis the ASES (68 to 89), SANE (64 to 84), and WOSI (65 to 81) scores improved significantly with 81% of participants returning to active duty and sports. A smaller civilian cohort of 11 patients saw similar improvements in functional outcome scores (ASES 54.5 to 78; SANE 42.5 to 70.4) and the 3 athletes returned to their sport [62]. While no study has specifically analyzed the outcomes of primary bicep tenodesis of overhead and throwing athletes, their results have been included in cohort studies [23, 25, 29, 30, 63]. Pogorzelski *et al* [63] reviewed 20 patients at an average follow up of 3.4 years and found that recreation athletes do benefit from primary subpectoral biceps tenodesis with 73% return to previous level including 80% of overhead athletes. Only two of the patients were throwers and two were volleyball players. Schöffl *et al* [64] demonstrated that non-throwing overhead athletes rock climbers have performed well with full return to sport following primary biceps tenodesis. Outcomes of elite throwers, however, following primary or revision bicep tenodesis have not been published to date. In fact a systematic review reported that only 71% of 49 studies of outcomes in baseball throwers with shoulder and elbow injuries reported return to play and 31% reported return to previous level [65]. As outlined earlier, the concern of glenohumeral stability without an intact biceps anchor has been a major concern [52], but a recent EMG and motion analysis study reports that pitchers regain physiologic neuromuscular control and normal pitching mechanics whether treated with SLAP repair or bicep tenodesis versus controls [66]. Chalmers *et al* [66] found that those with SLAP repairs had significantly different thoracic rotation movements compared to those with biceps tenodesis and controls. Another cadaveric study showed that bicep tenodesis had no detrimental effect on glenohumeral stability and repair of an anterior SLAP tear was the only intervention to restore translational stability [67]. Strauss *et al* including our senior author (BJC) concluded that bicep tenodesis is viable option for both primary and revision cases, but should be used with caution in overhead athletes. Despite these findings, Major League Baseball team surgeons still overwhelming favor repair over debridement or tenodesis [68].

Due to the results of biomechanical studies of the role of the biceps labral complex in glenohumeral stability [48-52] some have considered repair of SLAP lesion with a biceps tenodesis. Despite having similar return to sport or work, Chalmers *et al* [24] found that patients treated with combined procedures did significantly worse in terms of ASES and VAS scores. Even subgroup analysis with exclusion of worker’s compensation patients resulted in similar findings. At this time, the author recommends bicep tenodesis young patients in revision cases and in primary cases where the biceps is considered as a source of pain and selective injections into the biceps sheath temporarily relieves their pain.

### 4 OLDER, ATHLETIC PATIENTS

**Fig. (3):**
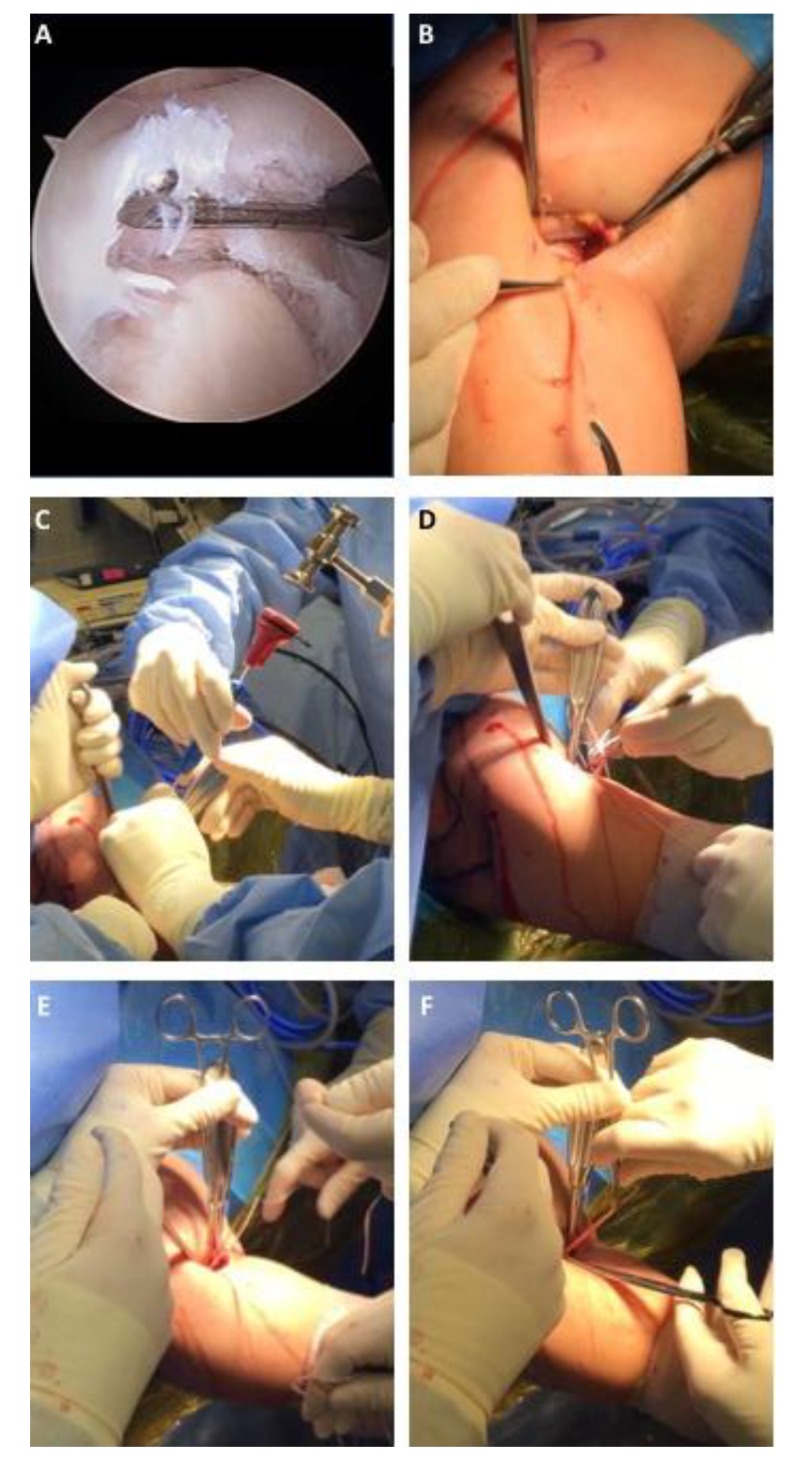
**Open All Suture Subpectoral Biceps Tenodesis for a Type II SLAP Tear.** Intraoperative images of a right shoulder depicting A) a Type II SLAP tear extending into the long head of the biceps, B) 3cm incision over the junction of the inferior border of the pectoralis major and anterior deltoid with the extracted long head of the biceps, C) placement of a suture anchor into the anterior humerus, D) placement of Krackow type sutures into the long head of the biceps, E) fixation of the long head of the biceps to the anterior humerus, and F) excision of the excess biceps tendon.

Numerous studies to date have linked the outcomes of SLAP repair versus tenodesis to the patient’s age [22, 23, 25-27, 35, 41, 61, 69]. The cohort studies of Alpert *et al* [22] and Schrøder *et al* [35] documented in the long-term, patients older and younger than forty years of age perform as well in terms of functional scores and outcomes. These studies however, discuss that the older patients were more inclined to get stiffer post operatively and took longer to regain full motion [22]. Katz *et al* [31] retrospectively looked at patients with poor outcomes, and the average age of the study was 43 years old. One of the largest cohort studies (179 patients), albeit a military population, found the relative risk for failure to be 3.45 in patients older than 36 years of age [34]. Taylor *et al* [69] performed a database study that demonstrated age greater than 40 (OR 1.5), Female Sex (OR 1.5), obesity (OR 1.8), smoking (OR 2.0) were all significant risk factors for failure following SLAP repair. Frank *et al* [70] analysis of a 62 patient cohort found similar risk factors for failure of SLAP repairs including age, smoking, diabetes, high demand labor and concomitant biceps symptoms. A systematic review pooled the studies and found that the rate of stiffness and post-operative complications increased with age leading the authors to recommend bicep tenodesis in patients over the age of forty [26]. This study also demonstrated that worker’s compensation is a risk factor for complications, and recommend bicep tenodesis in those cases as well. Denard *et al* [71] found similar findings that older patients with a SLAP repair had a delay in recovery of full motion compared to the tenodesis group. Retrospective studies have demonstrated equivalent outcomes between SLAP repair in younger patients and bicep tenodesis in older patients [23, 25, 29]. The cohort studies by Boileau *et al* [23] and Ek *et al* [25], had significantly older patients in the tenodesis group (37 versus 52; and 31 versus 47; respectively). These and other retrospective studies like Gottschalk *et al* [29] demonstrated that older patients may benefit from tenodesis instead of repair. The patients saw significantly better rates of return to sport following tenodesis. Boileau *et al* [23] had 93% satisfaction rate and 87% return to previous level of sport versus only 20% on the SLAP repair side. Bicep tenodesis allowed 89.66% of patients with either type II or Type IV lesions to return to their previous level of athletic activity [29]. Though not significant Ek *et al* [25] also saw a greater return to sport in the tenodesis versus repair groups (73% versus 60%; p=0.66). Due to the similar outcomes with fewer complications, the senior author recommends debridement and bicep tenodesis for older patients regardless of activity level and often in patients with third party liability or worker’s compensation cases (Fig. **3**). Notably, there is a trend toward the use of biceps tenodesis in younger patients as a primary treatment.

### 5 PATIENTS WITH CONCOMITANT PATHOLOGY

Superior labrum anterior to posterior tears often do not present in isolation [12, 13], and as a result patients with concomitant pathology should not only have the additional pathology addressed [72], but receive a bicep tenodesis. Gupta *et al* [30] retrospectively studied 28 patients with the average age of 43.7 years and concomitant bicep tendonitis and SLAP tear demonstrated significant improvements in ASES, SANE, SST, VAS, and SF-12 scores with excellent satisfaction in 80% of patients. A recent randomized control trial of patients with rotator cuff tears and labral-biceps lesions were broken into three treatment arms: debridement, tenotomy, and tenodesis. There was no difference in the outcome scores across all three groups in terms of range of motion and functional scores [33]. Franceschi *et al* [27] found patients with a rotator cuff tear in the presence of a SLAP tear who received bicep tenotomy performed better in terms improvement of UCLA scores (10.1 to 32.1) compared to the patients who received SLAP repair (10.4 to 27.9). Another cohort study also demonstrated greater improvement in function in terms of ASES (88.6 versus 80.4) and UCLA scores (29.6 versus 26.0) when patients underwent biceps tenotomy instead of slap repair when the patient had large to massive cuff tears [73]. In many of these studies the patient cohorts were older than 50 years of age, which could skew outcomes against SLAP repairs, however, we still recommend patients with concomitant pathology be treated with bicep tenodesis or tenotomy unless the patient is a young athlete or high demand patient.

When deciding between tenodesis and tenotomy in treatment of SLAP tears, the senior author recommends a thorough discussion with the patient and selective intraarticular and biceps tendon sheath injections to assess for primary pain generators. Hsu *et al* [39] performed a systematic review and found that tenodesis had less cosmetic deformity but increase chance of bicep pain when compared to patients who received tenotomy. The study recommended tenotomy for patients who were older, more overweight, low demand, non-worker’s compensation, and less concerned about cosmesis. Another systematic review of tenodesis versus tenotomy demonstrated equal excellent/good outcomes (74% versus 77%), an 8% versus 43% cosmetic deformity, and 24% versus 19% post-operative occurrence of bicipital pain [37]. We agree with Hsu *et al* [39] in that a discussion with the patient and a consideration of patient factors be considered in the final decision between tenodesis versus tenotomy. The senior author primarily relies upon biceps tenodesis to minimize the chance of postoperative deformity and the possibility of cramping and does so utilizing a sub-pectoral tenodesis using a suture anchor (Arthrex, Naples, FL, Fibertac, Biceps Anchor).

### 6 REVISION CASES

Risk factors for failure of SLAP repair include age, smoking, obesity, female sex, and concomitant bicep pathology [31, 38, 69, 70]. Katz *et al*. [31] found that once a patient has failed first time repair, 71% will fail conservative therapy and 32% will continue to have suboptimal outcomes after a second surgical intervention. Revision SLAP repair has limited data, but an 11 patient review of six overhead athletes and five workers compensation cases saw only improvement of ASES to 72.5 and return to work and sport of 57.8% and 42.2% respectively [74]. McCormick *et al* [61], however, demonstrated significant improvements in ASES, SANE, and WOSI scores in their retrospective review of bicep tenodesis of 42 patients with failed SLAP repairs, including an 81% return to sports. A smaller civilian cohort of 11 patients saw similar improvements in functional outcome scores (ASES 54.5 to 78; SANE 42.5 to 70.4) and the 3 athletes returned to their sport [62]. In the cohort published by Boileau *et al* [23] there were 4/10 patients in the repair group who were unsatisfied and underwent revision surgery to bicep tenodesis. All four patients had excellent outcomes and returned to sport. In agreement with previous studies [46, 60, 62], the senior author believes that bicep tenodesis is a reliable solution to failed SLAP repairs.

## CONCLUSION

SLAP tears can cause persistent pain and dysfunction in the shoulder and the management of Type II tears remains an evolving process with narrowing indications. Based on the existing literature, Type II tears in young (<40 years of age), athletic or high demand patients should be treated with direct SLAP repair. In older patients and worker’s compensation patients, Type II tears should often be treated with bicep tenodesis do to the higher rate of complications and revision/failures of repair in this population. In the cases of revision, bicep tenodesis remains an excellent solution for a difficult clinical scenario.

## DISCLOSOURE

Brian J. Cole reports the following disclosures: Aesculap/B.Braun: Research support; American Journal of Orthopedics: Editorial or governing board; American Journal of Sports Medicine: Editorial or governing board; Aqua Boom: Stock or stock Options; Arthrex, Inc: IP royalties, Paid consultant, Research support; Arthroscopy: Editorial or governing board; Arthroscopy Association of North America: Board or committee member; Athletico: Other financial or material support; Biomerix: Stock or stock Options; Cartilage: Editorial or governing board; DJ Orthopaedics: IP royalties; Elsevier Publishing: IP royalties; Flexion: Paid consultant; Geistlich: Research support; Giteliscope: Stock or stock Options; International Cartilage Repair Society: Board or committee member; Journal of Bone and Joint Surgery - American: Editor only: Editorial or governing board; Journal of Shoulder and Elbow Surgery: Editor only: Editorial or governing board; Journal of the American Academy of Orthopaedic Surgeons: Editor only: Editorial or governing board; JRF Ortho: Other financial or material support; National Institutes of Health (NIAMS & NICHD): Research support; Operative Techniques in Sports Medicine: Publishing royalties, financial or material support; Ossio: Stock or stock Options; Regentis: Paid consultant, Stock or stock Options; Sanofi-Aventis: Research support; Smith & Nephew: Other financial or material support; Paid consultant; Tornier: Other financial or material support; Zimmer: Paid consultant; Research support.

## References

[1] Andrews J.R., Carson W.G., McLeod W.D. (1985). Glenoid labrum tears related to the long head of the biceps.. Am. J. Sports Med..

[2] Snyder S.J., Karzel R.P., Del Pizzo W., Ferkel R.D., Friedman M.J. (1990). SLAP lesions of the shoulder.. Arthroscopy.

[3] Maffet M.W., Gartsman G.M., Moseley B. (1995). Superior labrum-biceps tendon complex lesions of the shoulder.. Am. J. Sports Med..

[4] Burkhart S.S., Morgan C.D. (1998). The peel-back mechanism: Its role in producing and extending posterior type II SLAP lesions and its effect on SLAP repair rehabilitation.. Arthroscopy.

[5] Keener J.D., Brophy R.H. (2009). Superior labral tears of the shoulder: pathogenesis, evaluation, and treatment.. J. Am. Acad. Orthop. Surg..

[6] Zhang A.L., Kreulen C., Ngo S.S., Hame S.L., Wang J.C., Gamradt S.C. (2012). Demographic trends in arthroscopic SLAP repair in the United States.. Am. J. Sports Med..

[7] Burkhart S.S., Morgan C.D., Kibler W.B. (2003). The disabled throwing shoulder: Spectrum of pathology Part I: Pathoanatomy and biomechanics.. Arthroscopy.

[8] Holtby R., Razmjou H. (2004). Accuracy of the Speed’s and Yergason’s tests in detecting biceps pathology and SLAP lesions: comparison with arthroscopic findings.. Arthroscopy.

[9] Cook C., Beaty S., Kissenberth M.J., Siffri P., Pill S.G., Hawkins R.J. (2012). Diagnostic accuracy of five orthopedic clinical tests for diagnosis of superior labrum anterior posterior (SLAP) lesions.. J. Shoulder Elbow Surg..

[10] Sodha S., Srikumaran U., Choi K., Borade A.U., McFarland E.G. (2017). Clinical Assessment of the Dynamic Labral Shear Test for Superior Labrum Anterior and Posterior Lesions.. Am. J. Sports Med..

[11] Parentis M.A., Glousman R.E., Mohr K.S., Yocum L.A. (2006). An evaluation of the provocative tests for superior labral anterior posterior lesions.. Am. J. Sports Med..

[12] Kim T.K., Queale W.S., Cosgarea A.J., McFarland E.G. (2003). Clinical features of the different types of SLAP lesions: An analysis of one hundred and thirty-nine cases.. J. Bone Joint Surg. Am..

[13] Snyder S.J., Banas M.P., Karzel R.P. (1995). An analysis of 140 injuries to the superior glenoid labrum.. J. Shoulder Elbow Surg..

[14] Mohtadi N.G., Vellet A.D., Clark M.L., Hollinshead R.M., Sasyniuk T.M., Fick G.H., Burton P.J. (2004). A prospective, double-blind comparison of magnetic resonance imaging and arthroscopy in the evaluation of patients presenting with shoulder pain.. J. Shoulder Elbow Surg..

[15] Rao A.G., Kim T.K., Chronopoulos E., McFarland E.G. (2003). Anatomical variants in the anterosuperior aspect of the glenoid labrum: a statistical analysis of seventy-three cases.. J. Bone Joint Surg. Am..

[16] Onyekwelu I., Khatib O., Zuckerman J.D., Rokito A.S., Kwon Y.W. (2012). The rising incidence of arthroscopic superior labrum anterior and posterior (SLAP) repairs.. J. Shoulder Elbow Surg..

[17] Weber S.C., Martin D.F., Seiler J.G., Harrast J.J. (2012). Superior labrum anterior and posterior lesions of the shoulder: Incidence rates, complications, and outcomes as reported by American Board of Orthopedic Surgery. Part II candidates.. Am. J. Sports Med..

[18] Patterson B.M., Creighton R.A., Spang J.T., Roberson J.R., Kamath G.V. (2014). Surgical trends in the treatment of superior labrum anterior and posterior lesions of the shoulder: Analysis of data from the american board of orthopaedic surgery certification examination database.. Am. J. Sports Med..

[19] Erickson B.J., Jain A., Abrams G.D., Nicholson G.P., Cole B.J., Romeo A.A., Verma N.N. (2016). SLAP Lesions: Trends in Treatment.. Arthroscopy.

[20] Werner B.C., Holzgrefe R.E., Brockmeier S.F. (2016). Arthroscopic surgical techniques for the management of proximal biceps injuries.. Clin. Sports Med..

[21] Brockmeyer M., Tompkins M., Kohn D.M., Lorbach O. (2016). SLAP lesions: A treatment algorithm.. Knee Surg. Sports Traumatol. Arthrosc..

[22] Alpert J.M., Wuerz T.H., O’Donnell T.F., Carroll K.M., Brucker N.N., Gill T.J. (2010). The effect of age on the outcomes of arthroscopic repair of type II superior labral anterior and posterior lesions.. Am. J. Sports Med..

[23] Boileau P., Parratte S., Chuinard C., Roussanne Y., Shia D., Bicknell R. (2009). Arthroscopic treatment of isolated type II SLAP lesions: biceps tenodesis as an alternative to reinsertion.. Am. J. Sports Med..

[24] Chalmers P.N., Monson B., Frank R.M., Mascarenhas R., Nicholson G.P., Bach B.R., Verma N.N., Cole B.J., Romeo A.A. (2016). Combined SLAP repair and biceps tenodesis for superior labral anterior-posterior tears.. Knee Surg. Sports Traumatol. Arthrosc..

[25] Ek E.T., Shi L.L., Tompson J.D., Freehill M.T., Warner J.J. (2014). Surgical treatment of isolated type II superior labrum anterior-posterior (SLAP) lesions: repair versus biceps tenodesis.. J. Shoulder Elbow Surg..

[26] Erickson J., Lavery K., Monica J., Gatt C., Dhawan A. (2015). Surgical treatment of symptomatic superior labrum anterior-posterior tears in patients older than 40 years: a systematic review.. Am. J. Sports Med..

[27] Franceschi F., Longo U.G., Ruzzini L., Rizzello G., Maffulli N., Denaro V. (2008). No advantages in repairing a type II superior labrum anterior and posterior (SLAP) lesion when associated with rotator cuff repair in patients over age 50: A randomized controlled trial.. Am. J. Sports Med..

[28] Friel N.A., Karas V., Slabaugh M.A., Cole B.J. (2010). Outcomes of type II superior labrum, anterior to posterior (SLAP) repair: Prospective evaluation at a minimum two-year follow-up.. J. Shoulder Elbow Surg..

[29] Gottschalk M.B., Karas S.G., Ghattas T.N., Burdette R. (2014). Subpectoral biceps tenodesis for the treatment of type II and IV superior labral anterior and posterior lesions.. Am. J. Sports Med..

[30] Gupta A.K., Chalmers P.N., Klosterman E.L., Harris J.D., Bach B.R., Verma N.N., Cole B.J., Romeo A.A. (2015). Subpectoral biceps tenodesis for bicipital tendonitis with SLAP tear.. Orthopedics.

[31] Katz L.M., Hsu S., Miller S.L., Richmond J.C., Khetia E., Kohli N., Curtis A.S. (2009). Poor outcomes after SLAP repair: Descriptive analysis and prognosis.. Arthroscopy.

[32] Kim S.H., Ha K.I., Kim S.H., Choi H.J. (2002). Results of arthroscopic treatment of superior labral lesions.. J. Bone Joint Surg. Am..

[33] Oh J.H., Lee Y.H., Kim S.H., Park J.S., Seo H.J., Kim W., Park H.B. (2016). Comparison of Treatments for Superior Labrum-Biceps Complex Lesions With Concomitant Rotator Cuff Repair: A Prospective, Randomized, Comparative Analysis of Debridement, Biceps Tenotomy, and Biceps Tenodesis.. Arthroscopy.

[34] Provencher M.T., McCormick F., Dewing C., McIntire S., Solomon D. (2013). A prospective analysis of 179 type 2 superior labrum anterior and posterior repairs: outcomes and factors associated with success and failure.. Am. J. Sports Med..

[35] Schrøder C.P., Skare O., Gjengedal E., Uppheim G., Reikerås O., Brox J.I. (2012). Long-term results after SLAP repair: A 5-year follow-up study of 107 patients with comparison of patients aged over and under 40 years.. Arthroscopy.

[36] Schrøder C.P., Skare Ø., Reikerås O., Mowinckel P., Brox J.I. (2017). Sham surgery versus labral repair or biceps tenodesis for type II SLAP lesions of the shoulder: a three-armed randomised clinical trial.. Br. J. Sports Med..

[37] Slenker N.R., Lawson K., Ciccotti M.G., Dodson C.C., Cohen S.B. (2012). Biceps tenotomy versus tenodesis: Clinical outcomes.. Arthroscopy.

[38] Cancienne J.M., Brockmeier S.F., Werner B.C. (2016). Tobacco use is associated with increased rates of infection and revision surgery after primary superior labrum anterior and posterior repair.. J. Shoulder Elbow Surg..

[39] Hsu A.R., Ghodadra N.S., Provencher M.T., Lewis P.B., Bach B.R. (2011). Biceps tenotomy versus tenodesis: A review of clinical outcomes and biomechanical results.. J. Shoulder Elbow Surg..

[40] Kibler W.B., Sciascia A. (2016). Current Practice for the Surgical Treatment of SLAP Lesions: A Systematic Review.. Arthroscopy.

[41] Waterman B.R., Cameron K.L., Hsiao M., Langston J.R., Clark N.J., Owens B.D. (2015). Trends in the diagnosis of SLAP lesions in the US military.. Knee Surg. Sports Traumatol. Arthrosc..

[42] Erickson B.J., Jain A., Cvetanovich G.L., Nicholson G.P., Cole B.J., Romeo A.A., Verma N.N. (2017). Biceps tenodesis: An evolution of treatment.. Am. J. Orthop..

[43] Edwards S.L., Lee J.A., Bell J.E., Packer J.D., Ahmad C.S., Levine W.N., Bigliani L.U., Blaine T.A. (2010). Nonoperative treatment of superior labrum anterior posterior tears: Improvements in pain, function, and quality of life.. Am. J. Sports Med..

[44] Fedoriw W.W., Ramkumar P., McCulloch P.C., Lintner D.M. (2014). Return to play after treatment of superior labral tears in professional baseball players.. Am. J. Sports Med..

[45] Jang S.H., Seo J.G., Jang H.S., Jung J.E., Kim J.G. (2016). Predictive factors associated with failure of nonoperative treatment of superior labrum anterior-posterior tears.. J. Shoulder Elbow Surg..

[46] Virk M.S., Tilton A.K., Cole B.J. (2015). Biceps tenodesis and superior labrum anterior to posterior (SLAP) tears.. Am. J. Orthop..

[47] Szabó I., Boileau P., Walch G. (2008). The proximal biceps as a pain generator and results of tenotomy.. Sports Med. Arthrosc. Rev..

[48] Pagnani M.J., Deng X.H., Warren R.F., Torzilli P.A., Altchek D.W. (1995). Effect of lesions of the superior portion of the glenoid labrum on glenohumeral translation.. J. Bone Joint Surg. Am..

[49] McMahon P.J., Burkart A., Musahl V., Debski R.E. (2004). Glenohumeral translations are increased after a type II superior labrum anterior-posterior lesion: a cadaveric study of severity of passive stabilizer injury.. J. Shoulder Elbow Surg..

[50] Panossian V.R., Mihata T., Tibone J.E., Fitzpatrick M.J., McGarry M.H., Lee T.Q. (2005). Biomechanical analysis of isolated type II SLAP lesions and repair.. J. Shoulder Elbow Surg..

[51] Youm T., ElAttrache N.S., Tibone J.E., McGarry M.H., Lee T.Q. (2009). The effect of the long head of the biceps on glenohumeral kinematics.. J. Shoulder Elbow Surg..

[52] McGarry M.H., Nguyen M.L., Quigley R.J., Hanypsiak B., Gupta R., Lee T.Q. (2016). The effect of long and short head biceps loading on glenohumeral joint rotational range of motion and humeral head position.. Knee Surg. Sports Traumatol. Arthrosc..

[53] Mihata T., McGarry M.H., Tibone J.E., Fitzpatrick M.J., Kinoshita M., Lee T.Q. (2008). Biomechanical assessment of Type II superior labral anterior-posterior (SLAP) lesions associated with anterior shoulder capsular laxity as seen in throwers: a cadaveric study.. Am. J. Sports Med..

[54] Youm T., Tibone J.E., ElAttrache N.S., McGarry M.H., Lee T.Q. (2008). Simulated type II superior labral anterior posterior lesions do not alter the path of glenohumeral articulation: a cadaveric biomechanical study.. Am. J. Sports Med..

[55] Sayde W.M., Cohen S.B., Ciccotti M.G., Dodson C.C. (2012). Return to play after Type II superior labral anterior-posterior lesion repairs in athletes: a systematic review.. Clin. Orthop. Relat. Res..

[56] Ide J., Maeda S., Takagi K. (2005). Sports activity after arthroscopic superior labral repair using suture anchors in overhead-throwing athletes.. Am. J. Sports Med..

[57] Morgan C.D., Burkhart S.S., Palmeri M., Gillespie M. (1998). Type II SLAP lesions: Three subtypes and their relationships to superior instability and rotator cuff tears.. Arthroscopy.

[58] Gorantla K., Gill C., Wright R.W. (2010). The outcome of type II SLAP repair: A systematic review.. Arthroscopy.

[59] Smith R., Lombardo D.J., Petersen-Fitts G.R., Frank C., Tenbrunsel T., Curtis G., Whaley J., Sabesan V.J. (2016). Return to play and prior performance in major league baseball pitchers after repair of superior labral anterior-posterior tears.. Orthop. J. Sports Med..

[60] Griffin J.W., Leroux T.S., Romeo A.A. (2017). Management of proximal biceps pathology in overhead athletes: What is the role of biceps tenodesis?. Am. J. Orthop..

[61] McCormick F., Nwachukwu B.U., Solomon D., Dewing C., Golijanin P., Gross D.J., Provencher M.T. (2014). The efficacy of biceps tenodesis in the treatment of failed superior labral anterior posterior repairs.. Am. J. Sports Med..

[62] Gupta A.K., Bruce B., Klosterman E.L., McCormick F., Harris J., Romeo A.A. (2013). Subpectoral biceps tenodesis for failed type II SLAP repair.. Orthopedics.

[63] Pogorzelski J., Horan M.P., Hussain Z.B., Vap A., Fritz E.M., Millett P.J. (2017). Subpectoral biceps tenodesis for treatment of isolated type II SLAP lesions in a young and active population.. Arthroscopy.

[64] Schöffl V., Popp D., Dickschass J., Küpper T. (2011). Superior labral anterior-posterior lesions in rock climbers—primary double tenodesis?. Clin. J. Sport Med..

[65] Makhni E.C., Saltzman B.M., Meyer M.A., Moutzouros V., Cole B.J., Romeo A.A., Verma N.N. (2017). Outcomes After Shoulder and Elbow Injury in Baseball Players: Are We Reporting What Matters?. Am. J. Sports Med..

[66] Chalmers P.N., Trombley R., Cip J., Monson B., Forsythe B., Nicholson G.P., Bush-Joseph C.A., Cole B.J., Wimmer M.A., Romeo A.A., Verma N.N. (2014). Postoperative restoration of upper extremity motion and neuromuscular control during the overhand pitch: evaluation of tenodesis and repair for superior labral anterior-posterior tears.. Am. J. Sports Med..

[67] Strauss E.J., Salata M.J., Sershon R.A., Garbis N., Provencher M.T., Wang V.M., McGill K.C., Bush-Joseph C.A., Nicholson G.P., Cole B.J., Romeo A.A., Verma N.N. (2014). Role of the superior labrum after biceps tenodesis in glenohumeral stability.. J. Shoulder Elbow Surg..

[68] Erickson B.J., Harris J.D., Fillingham Y.A., Cvetanovich G.L., Bush-Joseph C.A., Bach B.R., Romeo A.A., Verma N.N. (2016). Treatment of ulnar collateral ligament injuries and superior labral tears by major league baseball team physicians.. Arthroscopy.

[69] Taylor S.A., Degen R.M., White A.E., McCarthy M.M., Gulotta L.V., O’Brien S.J., Werner B.C. (2017). Risk factors for revision surgery after superior labral anterior-posterior repair: A national perspective.. Am. J. Sports Med..

[70] Frank R.M., Nho S.J., McGill K.C., Grumet R.C., Cole B.J., Verma N.N., Romeo A.A. (2013). Retrospective analysis of arthroscopic superior labrum anterior to posterior repair: Prognostic factors associated with failure.. Adv. Orthop..

[71] Denard P.J., Lädermann A., Parsley B.K., Burkhart S.S. (2014). Arthroscopic biceps tenodesis compared with repair of isolated type II SLAP lesions in patients older than 35 years.. Orthopedics.

[72] Mollon B., Mahure S.A., Ensor K.L., Zuckerman J.D., Kwon Y.W., Rokito A.S. (2016). Subsequent shoulder surgery after isolated arthroscopic SLAP repair.. Arthroscopy.

[73] Kim S.J., Lee I.S., Kim S.H., Woo C.M., Chun Y.M. (2012). Arthroscopic repair of concomitant type II SLAP lesions in large to massive rotator cuff tears: comparison with biceps tenotomy.. Am. J. Sports Med..

[74] Park S., Glousman R.E. (2011). Outcomes of revision arthroscopic type II superior labral anterior posterior repairs.. Am. J. Sports Med..

